# An exploratory model for the non-fatal drowning risks in children in Guangdong, China

**DOI:** 10.1186/s12889-019-6944-5

**Published:** 2019-05-17

**Authors:** Haofeng Xu, Xuhao Zhu, Zhishan Zhou, Yanjun Xu, Yongjian Zhu, Lifeng Lin, Jinying Huang, Ruilin Meng

**Affiliations:** 1 Guangdong Provincial Center for Disease Control and Prevention, Institute of Control and Prevention for Chronic Non-infective Disease, Guangzhou, China; 2 Qingyuan City Center for Disease Control and Prevention, Qingyuan, 511515 China; 3 Qingxin District Center for Disease Control and Prevention, Qingyuan, 511000 China; 4 Guangdong Provincial Center for Disease Control and Prevention, Center Director’s office, Guangzhou, China

**Keywords:** Drowning, Children, Risk factors, Logistic regression model, Prediction

## Abstract

**Background:**

Drowning is a leading cause of accidental death in children under 14 years of age in Guangdong, China. We developed a statistical model to classify the risk of drowning among children based on the risk factors.

**Methods:**

A multiple-stage cluster random sampling was employed to select the students in Grades 3 to 9 in two townships in Qingyuan, Guangdong. Questionnaire was a self-reported measure consisting of general information, knowledge, attitudes and activities. A univariate logistic regression model was used to preliminarily select the independent variables at a *P* value of 0.1 for multivariable model. Three-quarters of the participants were randomly selected as a training sample to establish the model, and the remaining were treated as a testing sample to validate the model.

**Results:**

A total of 8390 children were included in this study, about 12.18% (1013) experienced drowning during the past one year. In the univariate logistic regression model, introvert personality, unclear distributions of water areas on the way to school, and bad relationships with their classmates and families were positively associated with drowning. However, females, older age and lower swimming skills were negatively associated with drowning. After employing the prediction model with these factors to estimate drowning risk of the students in the testing samples, the results of Hosmer-Lemeshow tests showed non-significant differences between the predictive results and actual risk (χ2 = 5.97, *P* = 0.65).

**Conclusions:**

Male, younger children, higher swimming skills, bad relationship with their classmates and families, introvert personality and unclear distributions of water areas on the way to school were important risk factors of non-fatal drowning among children. The prediction model based on these variables has an acceptable predictive ability.

## Background

Drowning in children is a serious public health concern around the world, with an average of 372,000 death from drowning every year [1]. Drowning is a leading cause of accidental death in children under 14 years of age in China [2, 3] and in Guangdong Province located in southern China [4].

Although a series of intervention measures has been implemented in China, drowning in children remains an important public health issue [5, 6]. Most of the measures focused on risk factors of children and environment [1, 7–9], few studies have incorporated the characteristics of high risk children into the prevention strategy. We thus conducted this study to establish an exploratory model for predicting drowning risk among children.

We conducted this study in two rural townships in Qingyuan City of Guangdong Province because of the high mortality rate due of drowning and abundant natural and man-made open water bodies (unprotected nature or man-made water bodies). The findings from this study will provide important insights to the future prevention and control of drowning among children.

## Methods

### Subjects selection

Students were selected using multiple-stage cluster random sampling [10] in Guangdong Province in 2013. We randomly selected one city (Qingyuan City) from the overall 21 cities in Guangdong Province, and two townships from Qingyuan city because of numbers of rivers, ponds and reservoirs in the two townships. All the students in Grades 3 to 9 in the two townships were included in this survey. A total of 8966 students aged between 8 and 18 years were selected to participate in this study. All the investigators were trained prior to the survey to assist students completing the questionnaire in the classroom.

### Data collection and ethics considerations

This study was approved by the Ethics Review Committee of the National Center for Chronic and Noncommunicable Disease Control and Prevention, Chinese Center for Disease Control and Prevention (No: 201318). Agreement was obtained from every participant.

### Questionnaire

The questionnaire used in this study was developed based on our previous project of integrated intervention for prevention of non-fatal drowning in children in Guangdong Province, 2006–2008 [8, 11]. The variables included in the questionnaire were determined based on the literature and the current knowledge on the risk factors of drowning [12, 13]. The non-fatal drowning was defined, in accordance with the World Health Organization [1], as the experience of respiratory impairment from submersion/immersion in liquid among the alive students during the past one year before the survey.

We collected the potential drowning-related risk factors including general information (such as age, gender, etc), education levels of their parents, relationships between their family members, their own swimming skill levels, risk perception on drowning, high-risk behaviors (such as swimming in the pond, playing by the river, etc), environment (such as distance from school to open water, distance from home to open water, have open water on the way to school, etc) and disease burden on drowning (the cost of treating drowning, etc) [8, 9, 11, 14]. All the variables have been described in Table 1. All the questionnaires were checked by the investigators.

### Statistical analysis

The study participants were randomly divided into training and testing groups. The distributions of age, gender, and prevalence of drowning was similar in the two groups. We used training group to establish the model, and testing group to evaluate the model. Training group including three-quarters of all subjects was used to examine the significant variables of drowning and establish the prediction model. Testing group was used to evaluate the predictive effect of the model. Univariate logistic regression model was used to establish a predictive model by selecting significant variables of drowning. The receiver operating characteristic (ROC) curve and Hosmer-Lemeshow (H.L) Chi-square test were employed to test the sensitivity and specificity of the prediction model.

(1) **Variable selection:** We used univariate logistic regression model to examine the relationship between the potential risk factors and drowning, and select significant variables at a *P* value of 0.1 for multivariable logistic regression model [15, 16]. The prediction model was established based on the significant variables in the multivariable logistic regression model. (2) **Modeling evaluation:** We used the established prediction model to predict student’s drowning risk in the testing group, and evaluated the predictive effect of the prediction model by the area under the ROC curve (AUC) [17]. All the statistical analyses were conducted using the Stata software (version 13.0).

**Table 1:** Definitions and values of variables

	Definitions and values
Occurrence of drowning (y)*	No = 0, Yes = 1
Age (Years)(X1)	8≦X1 < 10 = 0, 10≦X1 < 12 = 1, 12≦X1 < 14 = 2, 14≦X1 < 18 = 3
Gender (X2)	Males = 0, Females = 1
Swimming skill (Meters) (X3)	≧100 = 0, 50–100 = 1, < 50 = 2, Unable to swim =3
Personality (X4)	Introvert = 0, Extrovert = 1, Between introvert and extrovert = 2, Do not know = 3
Relationships with classmates (X5)	Very good = 0, Good = 1, Not good = 2, Bad = 3
Relationships with family (X6)	Very good = 0, Good = 1, Not good = 2, Bad = 3
Number of siblings (X7)	One = 0, two or over = 1
Distance from school to open water (Meters) (X8)	< 100 = 0, 100–500 = 1, 500 + =2, None = 3, Do not know = 4
Distance from home to open water (Meters) (X9)	< 100 = 0, 100–500 = 1, 500 + =2, None = 3, Do not know = 4
Have open water on the way to school (X10)	Yes = 0, No = 1, Do not know = 2

*Occurrence of drowning: once had the experience of drowning = Yes; had no experience of drowning = No

## Results

### General characteristics of study subjects

A total of 8390 students were initially recruited in this study, after excluding 73 students due to the missing information, with 4367 males and 3950 females. The mean age was 12.43 ± 1.84 years. The risk of exposure to water activities (such as playing in or around open water) was 48.74%. A total of 1013 (12.18%) students experienced non-fatal drowning in the one year before the survey.

### Univariate analysis on the risk factors of drowning in children

Younger students, males, higher swimming skill, introvert personality, unclear distributions of water areas on the way to school, bad relationships with classmates and family were significantly associated with drowning risk in the univariate regression analyses (Table 2).

**Table 2:** Univariate analysis on the risk factors of drowning in children based on the training dataset

	Non-fatal drowning		*OR*	*95% CI*	*P*
	No n (%)	Yes n (%)			
Age (Years)(X1)					
8≦X1 < 10	1020(81.53)	231(18.47)	2.33	1.80~3.02	< 0.01
10≦X1 < 12	1609 (85.04)	283(14.96)	1.86	1.45~2.38	< 0.01
12≦X1 < 14	1056 (91.19)	102(8.81)	0.96	0.74~1.25	0.76
14≦X1 < 18	1774(91.58)	163(8.42)	1.00		
Gender(X2)					
Female	2686 (90.44)	284 (9.56)	0.72	0.61~0.85	< 0.01
Male	2773(84.85)	495(15.15)	1.00		
Swimming skill (Meters) (X3)					
≥100	575(80.65)	138(19.35)	1.97	1.54~2.52	< 0.01
50–100	1566(86.71)	240(13.29)	1.39	1.14~1.70	< 0.01
<50	948(85.95)	155(14.05)	1.44	1.15~1.80	< 0.01
Unable to swim	2370(90.60)	246(9.40)	1.00		
Personality (X4)					
Introvert	833(84.91)	148(15.09)	1.21	0.92~1.58	0.17
Extrovert	2846(87.30)	414(12.70)	1.11	0.88~1.39	0.39
Between introvert and extrovert	964(91.29)	92(8.71)	0.87	0.65~1.18	0.37
Do not know	816(86.72)	125(13.28)	1.00		
Relationships with classmates (X5)					
Very good	2725(88.30)	361(11.70)	0.53	0.34~0.82	< 0.01
Good	2518(88.10)	340(11.90)	0.54	0.35~0.84	< 0.01
Not good	124(74.70)	42(25.30)	1.06	0.61~1.85	0.83
Bad	92(71.88)	36(28.13)	1.00		
Relationships with family (X6)					
Very good	3859(88.55)	499(11.45)	0.26	0.15~0.45	< 0.01
Good	1422(87.03)	212(12.97)	0.32	0.19~0.56	< 0.01
Not good	138(77.09)	41(22.91)	0.54	0.29~1.03	0.06
Bad	40(59.70)	27(40.30)	1.00		
Number of siblings (X7)					
None	476(85.46)	81(14.54)	1.09	0.89~1.46	0.12
One or more	4983(87.71)	698(12.29)	1.00		
Distance from school to open water (Meters) (X8)					
<100	1162(84.94)	206(15.06)	1.53	1.23~1.90	< 0.01
100–500	744(87.63)	105(12.37)	1.30	1.00~1.69	< 0.05
500+	895(88.00)	122(11.99)	1.23	0.96~1.58	0.11
Have no water area	896(86.99)	134(13.01)	1.31	1.03~1.67	< 0.05
Do not know	1762(89.26)	212(10.74)	1.00		
Distance from home to open water (Meters) (X9)					
<100	1372(88.00)	187(11.99)	0.98	0.77~1.31	0.68
100–500	1018(86.71)	156(13.29)	1.04	0.81~1.38	0.62
500+	840(86.42)	132(13.58)	1.05	0.83~1.41	0.52
Have no water area	1218(88.45)	159(11.55)	0.97	0.74~1.29	0.46
Do not know	1011(87.46)	145(12.54)	1.00		
Have an open water on the way to school (X10)					
Yes	2009(87.92)	276(12.08)	0.69	0.56~0.86	< 0.01
No	2492(88.97)	309(11.03)	0.61	0.50~0.75	< 0.01
Do not know	958(83.16)	194(16.84)	1.00		

### Multivariate analysis on the risk factors of drowning in children

Drowning risk was significantly lower in females, students with older age and lower swimming skills. However, introvert personality, unclear distributions of water areas on their way to school, bad relationships with their classmates and families could significantly increase drowning risk in the multivariate analyses. In the final identified regression model, trend tests were also performed by including ordinal variables as continuous measures (Table 3).

**Table 3:** Multivariate analysis on the risk factors of drowning in children based on the training dataset

	*OR*	*95% CI*	*P* value	P for trend^*^
Age (X1)				
8≦X1 < 10	2.42	1.63~3.58	< 0.01	< 0.01
10≦X1 < 12	2.35	1.97~2.80	< 0.01	
12≦X1 < 14	1.45	1.21~1.74	< 0.01	
14≦X1 < 18	1.00			
Gender (X2)				
Male	1.44	1.24~1.66	< 0.01	
Female	1.00			
Swimming skill level (X3)				
≧100 m	2.12	1.72~2.62	< 0.01	< 0.01
50–100 m	1.42	1.19~1.69	< 0.01	
< 50 m	1.48	1.22~1.79	< 0.01	
Unable to swim	1.00			
Personality (X4)				
Introvert	1.26	1.00~1.59	< 0.05	
Extrovert	1.12	0.92~1.37	> 0.05	
Between intro and extro	0.84	0.65~1.09	> 0.05	
Do not know	1.00			
Relationships with classmates (X5)				
Very good	0.57	0.39~0.84	< 0.01	0.07
Good	0.59	0.40~0.88	< 0.01	
Not good	1.03	0.63~1.67	> 0.05	
Bad	1.00			
Relationships with family (X6)				
Very good	0.27	0.17~0.44	< 0.01	< 0.01
Good	0.34	0.21~0.56	< 0.01	
Not good	0.54	0.31~0.95	< 0.05	
Bad	1.00			
Have open water on the way to school (X10)				
Yes	0.76	0.63~0.91	< 0.01	
No	0.69	0.58~0.82	< 0.01	
Do not know	1.00			

^*^ Only for variables with three or more levels

The prediction model for drowning risk in children was established based on the variables of multivariate analysis.

**Fig. 1:**
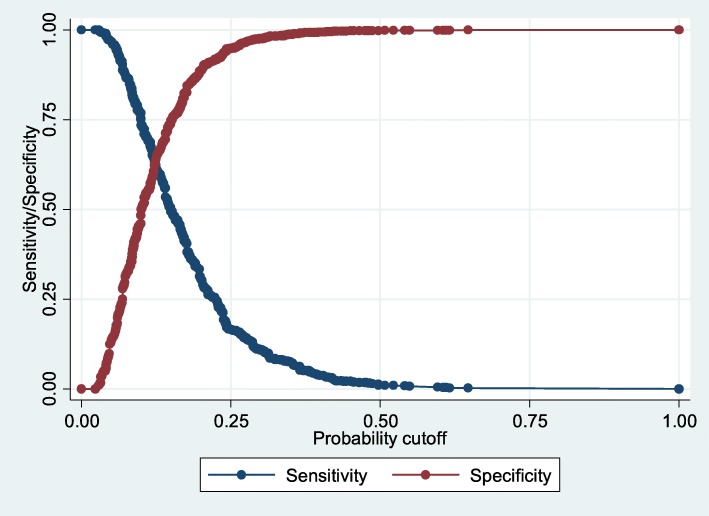
Sensitivity, specificity and cutoff point of drowning risks in the training dataset with drowning probability of 0.15 as a cutoff point

The drowning probability of 0.15 was set as a cutoff point. Students with drowning probability equal to or larger than 0.15 were defined as high drowning risk group, and students with drowning probability lower than 0.15 were categorized as low drowning risk group (Fig. 1).

### Predictive results and evaluation of the prediction model of drowning risks

**Fig. 2:**
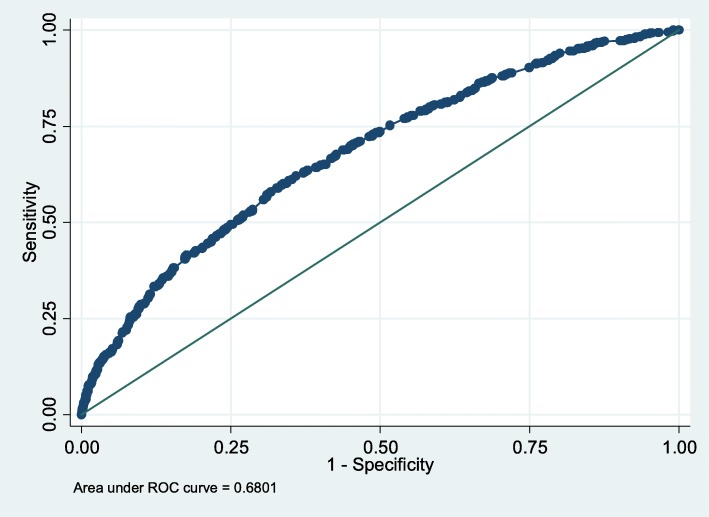
Evaluation of the predictive ability of drowning risk prediction model in the test dataset

We employed the prediction model to estimate drowning risk of the students in the testing group, and further tested the predictive results. The results of Hosmer-Lemeshow tests showed non-significant differences between the predictive results and actual risk (χ^2^ = 5.97, *P* = 0.65), indicating a high goodness of fit of the model. The sensitivity of the model was 49.44%, specificity was 74.76%, and the AUC was 0.680 (95% CI: 0.66~0.78), indicating that the model fits the data well (Fig. 2).

## Discussion

A number of epidemiological studies have revealed that drowning in children was affected by various risk factors including individual (male, decreasing age, etc), family (poverty, the education of their parents, etc) and social factors (less policies, abundant bodies of water, etc) [5, 8]. Although considerable efforts have been made to prevent drowning in children by Chinese governments, for example, the education department had released relevant documents, and developed education materials in the past years, drowning is still an important concern in children. In this study, we established a prediction model based on the risk factors of drowning in children.

The established model could divide students into high-risk and low-risk groups according to the drowning risks in children. We should pay more attention to the high-risk group when we run integrated intervention for prevention of children drowning.

The multivariate regression model is a widely-used method to establish a risk prediction model [18]. It can use cross-sectional investigation data to explore the population level risks, and can also predict the individual level risk of a certain disease using the cohort study data. The dependent variable in the regression model can be a binary variable, and independent variables can be either continuous variables or categorical variables. It is currently very common to establish the mathematical model based on both unconditional logistic regression model and long-term follow-up study data [19]. The earliest prediction model predicting the 10-year risk of diabetes in non-diabetes patients was established by Framingham Offspring who used the unconditional logistic regression model [20]. Since then, more and more studies employed a similar method [21, 22].

Based on our previous studies [8, 11, 23], we preliminarily selected ten variables such as age, gender, and relationships with classmates significant in the univariate analysis, seven variables of which (except for the variable of “distance from home to open water”, “number of sibling” and “distance from school to open water”) were found to have significant associations with drowning risk in the multivariate model. Age and gender are the common drowning risk factors in children in different countries [8, 11, 24]. We observed a significant association between higher swimming skill and increased risk of drowning in children, which is inconsistent with one previous study from Bangladesh [25]. Swimming ability could provide protection to children in some controlled environments such as in a swimming pool [26]. However, the effectiveness of swimming ability on reducing drowning risks has not been well defined. In contrast, individuals with better swimming skills might have more water exposures and dangerous behaviors such as swimming in natural collections of water and unsupervised water, which could increase their drowning risks [27]. The above may explain the difference between the protective factor in Bangladesh and risk factor in Guangdong province, China [25, 28]. We speculated that the living environment was different in Bangladesh (most households located near bodies of water) and in China (children need to spend several minutes or more to access to the bodies of water). Children with high level of swimming skills would like to play in or around water in China.

In the present study, we established a prediction model including seven independent variables based on the binary unconditional logistic regression model. According to the cutoff point, all the included children were divided into two groups. The test results in the test sample indicated that the model in predicting the risk of non-fatal drowning in students was acceptable, with an area under the ROC curve (AUC) of 0.680. Generally speaking, the AUC is around 0.7 in the population-based prediction models of a certain event [29, 30], and the AUC is usually larger than 0.85 in prediction models of clinical trials [31, 32].

A few limitations should be noted. The cross-sectional study has limitation in ascertaining causal relationship between the risk factors and drowning. Recall bias may exist in the cross-sectional study because most of the variables were measured by self-report, especially occurrence of drowning, the personality of the participants, the relationships with classmates and family. As one important factor associated with the risk of drowning, the information of water exposure was just simply collected in this survey. This might be the underlying reason for the higher drowning rate among the students with higher swimming skill observed in this study, this issue will be further investigated in future studies.

## Conclusion

In summary, male, younger children, higher swimming skills, bad relationship with their classmates and families, introvert personality and unclear distributions of water areas on their ways to school might be important risk factors of non-fatal drowning among children, and the prediction model based on these variables has an acceptable predictive ability.

## Abbreviations

H.L: Hosmer-Lemeshow
ROC: Receiver operating characteristic
UC: Area under the curve
WHO: World Health Organization
